# Culture Patterns of Biliary Stents in Patients With Periampullary Neoplasms After Endoscopic Retrograde Cholangiopancreatography (ERCP)

**DOI:** 10.7759/cureus.111075

**Published:** 2026-06-18

**Authors:** Loukas Rentifis, Angeliki Arapaki, Dimitrios Vouros, Konstantinos Bramis, Nikolaos Alexakis, George C Zografos, Konstantinos Toutouzas

**Affiliations:** 1 First Propaedeutic Department of Surgery, Hippocratio General Hospital, National and Kapodistrian University of Athens, School of Medicine, Athens, GRC; 2 Second Department of Surgery, Aretaieio University Hospital, National and Kapodistrian University of Athens, School of Medicine, Athens, GRC

**Keywords:** complications, implications, microbial, pancreaticoduodenectomy, positive for pathogens, preoperative biliary drainage, retrograde cholangiopancreatography, stent cultures

## Abstract

Preoperative biliary drainage (PBD) through endoscopic retrograde cholangiopancreatography (ERCP) is advocated to reduce complications following pancreaticoduodenectomy. However, randomized trials have provided inconclusive results. This study aimed to investigate the microflora colonizing preoperatively placed biliary stents in patients with neoplasms and their resistance to antibiotic treatment.

Data from 313 patients who underwent pancreaticoduodenectomy for neoplasms between 2010 and 2022 were analyzed. Among them, 171 patients underwent preoperative stent drainage, with 115 stents subjected to microbial culture. The stents were predominantly plastic.

Microbial analysis revealed that 85.2% of the stents were positive for pathogens, with Gram-negative bacteria being the most prevalent (63%). Fungi were isolated in 37.4% of cultures. *Klebsiella pneumoniae* exhibited multidrug resistance in 69.4% of cases, and pan-resistant *Pseudomonas aeruginosa* was found in three cultures.

The study found a significant association between waiting time to surgery and positive stent cultures. The presence of a stent increased the risk of changes in the microbiome. Interestingly, the presence of anaerobic strains was not detected in the sample. These findings suggest that biliary stents harbor a diverse range of microflora, which may have potential implications for postoperative outcomes, although direct clinical outcomes were not assessed in this study.

## Introduction

Preoperative biliary drainage (PBD) was first proposed by Whipple et al. in 1935 to reduce the complication rate following pancreaticoduodenectomy [1]. Theoretically, the preoperative correction of jaundice via endoscopic retrograde cholangiopancreatography (ERCP) should reduce postoperative morbidity and mortality. However, the results of randomized trials regarding its efficacy remain inconclusive. Several randomized trials have been conducted on patients with potentially reversible pancreatic cancer and malignant obstructive jaundice; one study suggested a benefit of PBD [2], five studies showed no benefit [3], and three studies yielded possibly negative results [4].

Biliary obstruction is associated with bacterial contamination of the bile and increased rate of septic complications after pancreaticoduodenectomy. Plastic stents are frequently used to maintain the patency of the bile duct, particularly in patients who are candidates for surgical treatment. However, the removal of the antimicrobial barrier of the sphincter of Oddi and the reduction of intraluminal pressure result in the reflux of duodenal contents. This leads to bacterial colonization of the stents and subsequent biofilm formation [5-8].

To address this, the primary objective of the present study was to perform descriptive microbiological profiling and evaluate the phenotypic antimicrobial resistance patterns of colonizing microflora in plastic biliary stents preoperatively placed via ERCP in patients with periampullary neoplasms. Furthermore, the secondary objective was to investigate whether specific preoperative timelines, clinical parameters, and demographic factors are independently associated with stent colonization and polymicrobial growth.

## Materials and methods

Ethical approval and study design

This study was designed as a retrospective analysis of a prospectively maintained database. The study protocol was conducted in strict adherence to the ethical guidelines established by the Declaration of Helsinki and was approved by the Institutional Review Board (IRB) of Hippocratio General Hospital in Athens (No.: 60/29-7-2021). Due to the retrospective nature of the analysis and the anonymization of data, the requirement for specific informed consent was waived by the IRB.

Study population

We reviewed the medical records of all patients who underwent pancreaticoduodenectomy (Whipple procedure) for suspected or confirmed pancreatic neoplasia at the First Department of Propaedeutic Surgery, National and Kapodistrian University of Athens, between January 2010 and December 2022.

A total of 313 patients were initially screened. Inclusion criteria for the final microbiological analysis were patients aged >18 years, histologically confirmed pancreatic neoplasia, and a history of PBD via ERCP. Patients with metallic stents or those whose stents were not retrieved intraoperatively were excluded. Out of 171 patients with PBD, 115 biliary stents were successfully retrieved and processed, constituting the final study cohort. All stents were plastic (polyethylene), straight or pigtail type, with diameters ranging from 7 to 10 Fr. The three primary reasons for non-retrieval were intraoperative technical difficulty in locating the stent due to anatomical variation or adhesions, stent migration identified at the time of surgery, and spontaneous passage prior to the operation. All patients received a single dose of prophylactic antibiotic (typically a second-generation cephalosporin) at the time of ERCP, as per international guidelines. Perioperative antibiotic prophylaxis (cefazolin 2 g IV) was also administered to all patients within 60 minutes before surgical incision. Information on therapeutic antibiotic courses administered between ERCP and surgery was not systematically recorded in our database.

Sample collection protocol

Stent retrieval was performed intraoperatively during the common bile duct (CBD) transection phase. To ensure the integrity of the microbiological analysis, a strict aseptic technique was employed. The stent was removed using sterile forceps, avoiding contact with the gastrointestinal lumen or skin, and immediately placed into a sterile, dry transport container. All samples were transported to the Department of Microbiology within 30 minutes of retrieval to ensure viability.

Microbiological analysis

Upon arrival at the laboratory, the "flush-culture" technique was utilized to harvest intraluminal biofilm. The stent lumen was catheterized using a sterile venous catheter, and 10 mL of sterile 0.9% normal saline was injected through the stent. The resulting broth was collected and inoculated into blood agar for the isolation of fastidious microorganisms, MacConkey agar for the selective isolation of Gram-negative enteric bacilli, and Columbia CNA agar for the selective isolation of Gram-positive cocci.

Cultures were incubated aerobically and anaerobically at 37°C for 48-72 hours; highly enriched anaerobic media specifically optimized for fastidious anaerobes were not used as part of the routine laboratory protocol at our institution during the study period. Bacterial identification and antimicrobial susceptibility testing (AST) were performed using semi-automated methods. We specifically screened for multidrug-resistant (MDR) phenotypes, including extended-spectrum beta-lactamases (ESBL), *Klebsiella pneumoniae* carbapenemase (KPC), and AmpC beta-lactamases. Microbial resistance was defined according to the minimum inhibitory concentration (MIC) breakpoints established by the European Committee on Antimicrobial Susceptibility Testing (EUCAST) guidelines [9]. In accordance with standardized international terminology, MDR was defined as non-susceptibility to at least one agent in three or more antimicrobial categories. Pandrug resistance (PDR) was defined as non-susceptibility to all agents in all available antimicrobial categories.

Positive cultures were analyzed for antimicrobial resistance on a per-isolate basis; thus, for polymicrobial cultures, susceptibility profiles were recorded independently for each isolated strain. 

Statistical analysis

Quantitative variables were tested for normal distribution using the Shapiro-Wilk test [10]. As the majority of continuous variables did not follow a normal distribution, they are presented as median values with interquartile ranges (IQR, 75th-25th percentile). The non-parametric Mann-Whitney U test was employed to compare distributions across bivariate categories. Qualitative variables are reported as absolute frequencies (N) and relative frequencies (%). Associations between qualitative variables were examined using Pearson's chi-square test or Fisher's exact test, as appropriate. No proprietary scoring systems or licensed clinical assessment tools were used in this study. All laboratory definitions and antimicrobial susceptibility interpretations were based on publicly available EUCAST guidelines [9]. All statistical tests were two-sided, with statistical significance defined as p < 0.05. Data analysis was performed using IBM SPSS Statistics for Windows, Version 25 (Released 2017; IBM Corp., Armonk, New York, United States) [11]. Analysis was performed under the institutional license of the National and Kapodistrian University of Athens.

## Results

Demographic and clinical characteristics

Baseline demographic and clinical characteristics of the study population are summarized in Table 1. The study sample consisted of 115 patients with a median age of 67 years (IQR: 14). The majority of patients were male (58.3%, n=67), and the median BMI was 25.8 kg/m² (IQR: 6). Median waiting time from preoperative ERCP to surgery was 20 days (IQR: 20), while the median duration of jaundice prior to drainage was 20 days. The median postoperative hospital stay was 12 days (IQR: 11). All patients underwent pancreaticoduodenectomy for neoplasms. The indications included pancreatic head malignancies (93.9%, n=108), distal cholangiocarcinomas, and isolated cases of neuroendocrine carcinomas (NEC). The median preoperative total bilirubin was 2.9 mg/dL (IQR: 5.0). Neoadjuvant therapy was administered to eight patients (7%).

**Table 1:** Baseline demographic, clinical, and perioperative characteristics of the study population. Continuous variables are presented as median (interquartile range, IQR). Categorical variables are presented as a number (percentage). BMI: body mass index; DM: diabetes mellitus; Τ.Bil: total bilirubin TNM staging according to the American Joint Committee on Cancer (AJCC) Cancer Staging Manual, 8th edition [12].

Variable	Summary statistics
Age (ys) (median (IQR))	67 (14)
Post-surgery length of stay (days) (median (IQR))	12 (11)
CA 19.9 (Pre-surgery) (median (IQR))	73.1 (373.3)
Τ.Bil (Pre-surgery) (median (IQR))	2.9 (5)
Wait time for surgery (days) (median (IQR))	22 (20)
Jaundice (days) (median (IQR))	20 (20)
BMI (kg/m^2^) (median (IQR))	25.8 (6)
Gender (N (%))	
Men	67 (58.3)
Women	48 (41.7)
Lesion type (N (%))	
Benign	7 (6.1)
Malignant	108 (93.9)
Stent culture (N (%))	
Negative	17 (14.8)
Positive	98 (85.2)
Gram + in stent culture (N (%))	
No	76 (66.1)
Yes	39 (33.9)
Gram - in stent culture (N (%))	
No	52 (45.2)
Yes	63 (54.8)
Fungi in stent culture (N (%))	
No	72 (62.6)
Yes	43 (37.4)
Number of bacteria in culture (N (%))	
Pure cultures	47 (43.5)
Mixed cultures	61 (56.5)
Blood culture result (N (%))	
Negative	41 (36.9)
Positive	70 (63.1)
Urine culture (N (%))	
No	53 (47.7)
Yes	58 (52.3)
Urine culture result (N (%))	
Negative	91 (91.9)
Positive	8 (8.1)
Neoadjuvant chemotherapy (N (%))	
No	107 (93)
Yes	8 (7)
Obstructive jaundice (N (%))	
No	4 (5.6)
Yes	67 (94.4)
Weight loss (N (%))	
No	13 (18.8)
Yes	56 (81.2)
Anorexia (N (%))	
No	47 (68.1)
Yes	22 (31.9)
DM (N (%))	
No	49 (71)
Yes	20 (29)
DM deterioration (N (%))	
No	56 (81.2)
Yes	13 (18.8)
Smoking (N (%))	
No	34 (49.3)
Yes	35 (50.7)
Alcohol (N (%))	
No	52 (75.4)
Yes	17 (24.6)
Cholecystectomy (N (%))	
No	59 (85.5)
Yes	10 (14.5)
T stage (N (%))	
0	4 (3.8)
1	7 (6.7)
2	31 (29.5)
3	59 (56.2)
4	4 (3.8)
N stage (N (%))	
0	31 (29.5)
1	60 (57.1)
2	14 (13.3)
M stage (N (%))	
0	100 (95.2)
1	5 (4.8)

Microbiological profile of biliary stents

Out of the 115 stents submitted for culture, 98 (85.2%) tested positive for a pathogen. Among the isolated pathogens, 63 were Gram-negative bacteria, and n=39 were Gram-positive bacteria. Fungal colonization was observed in 43 (37.4%) cultures. Polymicrobial (mixed) cultures were present in 56.5% of cases (Table 1).

A total of 172 microbial strains were isolated (Tables 2, 3). The distribution of microorganisms isolated from biliary stents is presented in Table 2. The most frequently identified microorganisms were *Candida* species (n=43), followed closely by *Klebsiella pneumoniae* (n=36) and *Escherichia coli *(n=27). The *Candida* species identified included *C. albicans* (the most prevalent), *C. glabrata, C. parapsilosis, *and* C. lusitaniae*. Among bacteria, *Klebsiella pneumoniae *and *E. coli *were the predominant isolates, followed by *Enterobacter cloacae, Pseudomonas aeruginosa*, and *Enterococcus *spp.

**Table 2:** Distribution of microorganisms isolated from biliary stent cultures. Values represent the total number of isolates recovered from all positive stent cultures. Polymicrobial cultures may contribute more than one isolate per patient.

Microorganism	Number
*Candida *spp.	43
Klebsiella pneumoniae	36
E.coli	27
*Enterococcus *spp*.*	26
Enterobacter cloacae/aeroginosa	13
Pseudomonas aeruginosa	7
Staphylococcus epidermidis	5
Proteus mirabilis	3
Citrobacter koseri	3
Acinetobacter baumanii	3
Serratia marcescens	2
Aeromonas hydrophila	1
Stenotrophomonas maltophilia	1
Streptococcus mitis	1
*Pantoea *spp*.*	1
Grand Total	172

**Table 3:** Antimicrobial susceptibility patterns of the most frequently isolated microorganisms. Antimicrobial susceptibility was determined according to the European Committee on Antimicrobial Susceptibility Testing (EUCAST) clinical breakpoints (version 12.0) [9]. Results are expressed as a number (percentage).

Microorganism	Category	N	%
*Klebsiella pneumoniae *(Antibiogram)	Sensitive	9	27.3%
	Resistant	24	72.7%
*Candida *spp*.* (Antibiogram)	Sensitive	23	63.9%
	Resistant	13	36.1%
*Enterobacter cloacae/aeroginosa* (Antibiogram)	Sensitive	5	50.0%
	Resistant	5	50.0%
*E. coli *(Antibiogram)	Sensitive	15	60.0%
	Resistant	10	40.0%
*Pseudomonas aeruginosa *(Antibiogram)	Sensitive	0	0.0%
	Resistant	6	100.0%
*Enterococcus *spp*.* (Antibiogram)	Sensitive	9	34.6%
	Resistant	17	65.4%

Factors associated with stent colonization

A comparison between patients with positive stent cultures and those with negative cultures is shown in Tables 4, 5. There were no statistically significant differences between the two groups regarding demographic characteristics or most microbiological parameters (p > 0.05). However, significant differences were observed regarding preoperative timelines and bilirubin levels. The waiting time to surgery was significantly longer in patients with positive stent cultures compared to those with negative cultures (p=0.010). Similarly, preoperative total bilirubin levels were significantly higher in the positive culture group (p=0.030).

**Table 4:** Comparison of categorical variables between patients with positive and negative biliary stent cultures. Categorical variables are presented as a number (percentage). Comparisons were performed using Pearson’s chi-square (χ²) test or Fisher’s exact test, as appropriate. All tests were two-sided. Pearson’s chi-square (χ²) test statistic is reported where applicable. For Fisher’s exact test, no test statistic is available. DM: diabetes mellitus 1: p-values from Pearson’s χ2 and Fisher’s exact tests

Stent culture							
		Negative		Positive			
		N	%	N	%	p^1^	Test statistic
Gender	Men	9	52.9%	58	59.2%	0.630	0.232
	Women	8	47.1%	40	40.8%		
Lesion type	Benign	0	0.0%	7	7.1%	0.592	
	Malignant	17	100.0%	91	92.9%		
Gram + in stent culture	No	17	100.0%	59	60.2%	0.001	
	Yes	0	0.0%	39	39.8%		
Gram - in stent culture	No	16	94.1%	36	36.7%	<0.001	
	Yes	0	5.9%	62	63.3%		
Fungi in stent culture	No	17	100.0%	55	56.1%	<0.001	
	Yes	0	0.0%	43	43.9%		
Number of bacteria in culture	Pure cultures	10	100.0%	37	37.8%	<0.001	
	Mixed cultures	0	0.0%	61	62.2%		
Blood culture result	Negative	6	37.5%	35	36.8%	0.960	0.003
	Positive	10	62.5%	60	63.2%		
Urine culture	No	9	56.3%	44	46.3%	0.462	0.542
	Yes	7	43.8%	51	53.7%		
Urine culture result	Negative	14	87.5%	77	92.8%	0.612	
	Positive	2	12.5%	6	7.2%		
Neoadjuvant chemotherapy	No	16	94.1%	91	92.9%	0.999	
	Yes	1	5.9%	7	7.1%		
Obstructive jaundice	No	1	6.3%	3	5.5%	0.999	
	Yes	15	93.8%	52	94.5%		
Weight loss	No	4	25.0%	9	17.0%	0.481	
	Yes	12	75.0%	44	83.0%		
Anorexia	No	11	68.8%	36	67.9%	0.999	0.004
	Yes	5	31.3%	17	32.1%		
DM	No	11	68.8%	38	71.7%	0.999	
	Yes	5	31.3%	15	28.3%		
DM deterioration	No	11	68.8%	45	84.9%	0.162	
	Yes	5	31.3%	8	15.1%		
Smoking	No	9	56.3%	25	47.2%	0.524	0.405
	Yes	7	43.8%	28	52.8%		
Alcohol	No	11	68.8%	41	77.4%	0.518	
	Yes	5	31.2%	12	22.6%		

**Table 5:** Comparison of continuous variables between patients with positive and negative biliary stent cultures. Comparisons between groups were performed using the Mann-Whitney U test. All tests were two-sided. Continuous variables are presented as median and interquartile range (IQR). * Statistically significant difference (p < 0.05) between positive and negative stent culture groups. 1: p-values from the Mann-Whitney U test; 2: Z values from the Mann-Whitney U test

Variable	Stent culture						
	Negative		Positive				
	Median	IQR	Median	Median difference (95% CI)	IQR	p1	Z value2
Age (ys)	68.0	8.0	66.0	-	15.0	307	-1.021
Post-surgery length of stay (days)	12.0	13.0	12.0	-	11.0	507	-664
CA 19.9 (Pre-surgery)	53.3	329.7	79.0	-	383.8	772	-290
Τ.Bil (Pre-surgery)	5.0	8.1	2.7	2.3(0.1–4.5)*	4.8	30	-2.171
Wait time for surgery (days)	10.00	2.00	27.50	17.5(4.0–31.0)	25.00	10	-2.579
Jaundice (days)	20.0	16.5	26.0	-	21.5	159	-1.408
BMI (kg/m^2^)	25.1	6.6	25.8		6.1	854	-184

Analysis of mixed cultures

When analyzing variables based on the number of microbes cultured (Tables 6, 7), the majority of cultures in both monomicrobial and polymicrobial groups tested positive for Gram-negative pathogens. Notably, smoking status was significantly associated with the likelihood of presenting a mixed culture (p < 0.05).

**Table 6:** Comparison of categorical demographic, clinical, and microbiological variables according to culture type (monomicrobial versus polymicrobial). Categorical variables are presented as a number (percentage), and were compared using Pearson’s chi-square (χ²) test or Fisher’s exact test, as appropriate. Pearson’s chi-square (χ²) test statistic is reported where applicable. For Fisher’s exact test, no test statistic is available. All tests were two-sided. DM: diabetes mellitus 1: p-values from χ2 test

Variable		Number of bacteria in culture					
		Pure cultures		Mixed cultures			
		N	%	N	%	p^1^	Test statistic
Gender	Men	25	53.2%	38	62.3%	0.341	0.905
	Women	22	46.8%	23	37.7%		
Lesion type	Benign	3	6.4%	4	6.6%	0.999	
	Malignant	44	93.6%	57	93.4%		
Stent culture	Negative	10	21.3%	0	0.0%	<0.001	
	Positive	37	78.7%	61	100.0%		
Gram + in stent culture	No	39	83.0%	30	49.2%	<0.001	13.144
	Yes	8	17.0%	31	50.8%		
Gram - in stent culture	No	31	66.0%	14	23.0%	<0.001	20.201
	Yes	16	34.0%	47	77.0%		
Fungi in stent culture	No	36	76.6%	29	47.5%	0.002	9.352
	Yes	11	23.4%	32	52.5%		
Blood culture result	Negative	20	43.5%	19	32.2%	0.235	1.407
	Positive	26	56.5%	40	67.8%		
Urine culture	No	23	50.0%	27	45.8%	0.667	0.186
	Yes	23	50.0%	32	54.2%		
Urine culture result	Negative	38	92.7%	49	94.2%	0.999	
	Positive	3	7.3%	3	5.8%		
Neoadjuvant chemotherapy	No	46	97.9%	54	88.5%	0.078	
	Yes	1	2.1%	7	11.5%		
Obstructive jaundice	No	0	0.0%	3	7.3%	0.290	
	Yes	24	100.0%	38	92.7%		
Weight loss	No	7	29.2%	4	10.3%	0.086	
	Yes	17	70.8%	35	89.7%		
Anorexia	No	19	79.2%	26	66.7%	0.286	1.138
	Yes	5	20.8%	13	33.3%		
DM	No	16	66.7%	29	74.4%	0.512	0.431
	Yes	8	33.3%	10	25.6%		
DM deterioration	No	17	70.8%	34	87.2%	0.185	
	Yes	7	29.2%	5	12.8%		
Smoking	No	16	66.7%	15	38.5%	0.030	4.729
	Yes	8	33.3%	24	61.5%		
Alcohol	No	19	79.2%	28	71.8%	0.514	0.426
	Yes	5	20.8%	11	28.2%		
Cholecystectomy	No	20	83.3%	34	87.2%	0.721	
	Yes	4	16.7%	5	12.8%		

**Table 7:** Comparison of continuous demographic, clinical, and microbiological variables according to culture type (monomicrobial versus polymicrobial). Continuous variables are presented as median (interquartile range, IQR) and were compared using the Mann-Whitney U test. All tests were two-sided. 1: p-values from the Mann-Whitney U test; 2: Z values from the Mann-Whitney U test

Variable	Pure cultures		Mixed cultures			
	Median	IQR	Median	IQR	p^1^	Z value^2^
Age (ys)	68.0	19.0	65.0	12.0	0.295	-1.048
Post-surgery length of stay (days)	13.0	13.0	11.0	8.0	0.136	-1.489
CA 19.9 (Pre-surgery)	97.6	593.8	61.5	192.9	0.139	-1.479
Τ.Bil (Pre-surgery)	3.7	8.9	2.1	4.2	0.024	-2.259
Wait time for surgery (days)	25.00	12.00	30.00	25.00	0.902	-0.123
Jaundice (days)	23.0	15.0	27.0	26.0	0.924	-0.095
BMI (kg/m^2^)	26.8	5.6	25.6	6.1	0.634	-0.476

Antimicrobial resistance

Resistance patterns for the most common microorganisms are detailed in Table 8. While most microorganisms demonstrated sensitivity to common antibiotics, specific MDR phenotypes were observed. *Klebsiella pneumoniae* isolates were MDR in 69.4% of cases, with 33.3% (n=12) producing KPC. Furthermore, PDR phenotypes were identified in 44.4% (n=16) isolates of *Klebsiella pneumoniae*. In the *Pseudomonas aeruginosa* group, a 100% resistance rate was noted in the antibiogram (n=6), with 33% (n=2) demonstrating a PDR phenotype. A substantial rate of resistance to quinolones was noted across the sample.

**Table 8:** Antimicrobial resistance phenotypes of isolated microorganisms. Resistance phenotypes were defined according to the European Committee on Antimicrobial Susceptibility Testing (EUCAST) clinical breakpoints (version 12.0) [9]. Values are expressed as a percentage (number). VRE: vancomycin-resistant Enterococcus; ESBL: extended-spectrum beta-lactamase; AmpC: AmpC beta-lactamase; MBL: metallo-beta-lactamase; KPC: *Klebsiella pneumoniae* carbapenemase; QR: quinolone-resistant; PDR: pan-drug resistant

Bacteria	Resistance pattern						
	KPC	ESBLs	AmpC	MBLs	QR	VRE	PDR
E. coli		14.8% (4)			11.1% (3)		
Klebsiella pneumoniae	33.3% (12)	5.6% (2)		33.3% (12)	55.6% (20)		44.4% (16)
Enterobacter cloacae/aeroginosa			70% (7)		10% (1)		20% (2)
Pseudomonas aeruginosa				42% (3)	33% (2)		33% (2)
*Enterococcus *spp*.*						20% (5)	

## Discussion

The colonization of endoscopically placed stents by various microflora during preoperative ERCP is a well-documented phenomenon. The predominant mechanism driving this colonization is the ascending primary inoculation of microorganisms from the duodenal flora, facilitated by the removal of the natural barrier of the sphincter of Oddi via sphincterotomy. The presence of a stent significantly amplifies this risk; Scheufele et al. observed that the microbiome was altered in 97.1% of stented patients compared to only 18.6% of patients without a stent [13]. Furthermore, the formation of a biofilm on the inner surface of the stent, composed of amorphous bile sludge, microbial byproducts, proteins, dietary fiber, and bile salts, leads to progressive narrowing of the stent lumen [14,15]. Additionally, the risk of pathogen transmission between patients using the same endoscope cannot be entirely excluded, despite rigorous antisepsis and hygiene protocols [16].

Polymicrobial growth in biliary stents is frequently reported in the literature, with rates ranging from 8% to 67% [15-18]. The present study confirmed this trend but observed a notably higher rate of 85.2%. This elevated rate may be attributed to the rigorous intraoperative sample collection method employed, which minimizes external contamination, as well as the inherent immunosuppression of the study population, the majority of whom suffered from malignant neoplasms.

Our analysis revealed that the presence of a positive stent culture was significantly associated with the waiting time before the surgical procedure (p=0.010). Specifically, a significantly higher proportion of positive results was observed during the 2011-2017 period, which likely corresponds to longer preoperative waiting times experienced during those years. Furthermore, the number of bacterial strains isolated per stent correlated significantly with the duration of stent placement (p=0.01), although the specific type of bacteria isolated did not show a correlation with stent duration (p > 0.05).

The microflora isolated from the biliary stents in this study consisted exclusively of aerobic microorganisms and fungi. In contrast to previous studies [15,16,19], no anaerobic strains were detected in our sample. This discrepancy may be due to the specific characteristics of our patient cohort, which consisted primarily of individuals with malignancies. Alternatively, the absence of anaerobes may reflect the technical challenges associated with their isolation and their high diversity. Previous research has indicated that anaerobes play an important role in stent occlusion and the pathogenesis of cholangitis [20,21].

Regarding the specific pathogens identified, earlier studies have established baseline microbial expectations in these cohorts [22]. A recent meta-analysis presented similar findings regarding bile pathogens [23]. However, a study by Lübbert et al. [24] described a microbial pattern with a higher proportion of Gram-positive bacteria and fungi, though their cohort consisted largely of patients with benign disease and long-dwelling stents, unlike the malignant cohort in the current study.

Blood cultures were not obtained systematically across the entire cohort; they were drawn solely at the clinical discretion of the treating physician for symptomatic patients (e.g., fever >38.0°C, rigors, or signs of sepsis/cholangitis post-ERCP). Consequently, the denominator for this analysis (n=111) comprises only patients with a direct clinical indication for testing. We agree that the 63.1% positivity rate must be interpreted in the context of this specific, symptomatic subgroup rather than as an overall prevalence.

This study has several limitations. Its retrospective, single-center design introduces potential selection bias, as 56 of 171 stents were unretrieved due to migration or anatomical reasons. The cohort predominantly comprised malignant cases (93.9%), limiting generalizability to benign biliary conditions. We lacked systematic documentation of individual pre-operative antibiotic exposures, which heavily influence resistance phenotypes. The saline "flush-culture" technique introduced oxidative stress, likely compromising the viability of fastidious anaerobes. The small negative-culture subgroup (n=17) and the absence of an a priori power calculation limit the statistical strength of our secondary analyses. Furthermore, our statistical analysis was limited to bivariate comparisons. Due to the retrospective nature of the study and sample size constraints, we did not perform multivariable adjustment for potential confounding factors. This absence of multivariable analysis should be taken into consideration when interpreting the associations presented. The absence of multivariable adjustment precludes definitive causal inference regarding the independent effect of any single clinical variable on stent colonization. We recommend that future prospective studies with larger sample sizes incorporate multivariable modeling to isolate independent predictors of biliary stent colonization. Finally, we did not directly correlate stent colonization patterns with postoperative surgical site infections or pancreatic fistulas, leaving these clinical implications hypothesis-generating.

## Conclusions

The study sample included the majority of patients with malignant diseases. The group of these patients has a long and complex course to surgery, so the results may differ from those of studies of patients with benign diseases and stents (cholangitis, lithiasis, etc.). While the high prevalence of resistant pathogens and fungi colonizing biliary stents highlights a significant clinical challenge, our study did not directly analyze the correlation between these microbiological findings and specific postoperative clinical outcomes, such as surgical site infections or sepsis. Therefore, inferences regarding the direct impact of stent colonization on postoperative septic complications must be drawn with caution, and further prospective studies are warranted to establish causality.
